# Common Infections and Antibiotic Prescribing during the First Year of the COVID-19 Pandemic: A Primary Care-Based Observational Cohort Study

**DOI:** 10.3390/antibiotics10121521

**Published:** 2021-12-13

**Authors:** Josi A. Boeijen, Alike W. van der Velden, Saskia Hullegie, Tamara N. Platteel, Dorien L. M. Zwart, Roger A. M. J. Damoiseaux, Roderick P. Venekamp, Alma C. van de Pol

**Affiliations:** Julius Center for Health Sciences and Primary Care, University Medical Center Utrecht, Utrecht University, Heidelberglaan 100, 3584 CX Utrecht, The Netherlands; a.w.vandervelden@umcutrecht.nl (A.W.v.d.V.); s.hullegie@umcutrecht.nl (S.H.); T.N.Platteel-3@umcutrecht.nl (T.N.P.); D.Zwart@umcutrecht.nl (D.L.M.Z.); R.A.M.J.Damoiseaux@umcutrecht.nl (R.A.M.J.D.); R.P.Venekamp@umcutrecht.nl (R.P.V.); A.C.vandePol-11@umcutrecht.nl (A.C.v.d.P.)

**Keywords:** COVID-19, pandemic, antibiotic, infectious disease, incidence, routine care data, complications

## Abstract

Presentation and antibiotic prescribing for common infectious disease episodes decreased substantially during the first COVID-19 pandemic wave in Dutch general practice. We set out to determine the course of these variables during the first pandemic year. We conducted a retrospective observational cohort study using routine health care data from the Julius General Practitioners’ Network. All patients registered in the pre-pandemic year (*n* = 425,129) and/or during the first pandemic year (*n* = 432,122) were included. Relative risks for the number of infectious disease episodes (respiratory tract/ear, urinary tract, gastrointestinal, and skin), in total and those treated with antibiotics, and proportions of episodes treated with antibiotics (prescription rates) were calculated. Compared to the pre-pandemic year, primary care presentation for common infections remained lower during the full first pandemic year (RR, 0.77; CI, 0.76–0.78), mainly attributed to a sustained decline in respiratory tract/ear and gastrointestinal infection episodes. Presentation for urinary tract and skin infection episodes declined during the first wave, but returned to pre-pandemic levels during the second and start of the third wave. Antibiotic prescription rates were lower during the full first pandemic year (24%) as compared to the pre-pandemic year (28%), mainly attributed to a 10% lower prescription rate for respiratory tract/ear infections; the latter was not accompanied by an increase in complications. The decline in primary care presentation for common infections during the full first COVID-19 pandemic year, together with lower prescription rates for respiratory tract/ear infections, resulted in a substantial reduction in antibiotic prescribing in Dutch primary care.

## 1. Introduction

The WHO declared COVID-19 a global pandemic in March 2020 [1], with three consecutive pandemic waves in the Netherlands, the first of which lasted from March 2020 through May 2020. Thereafter, a period of relatively low COVID-19 incidence followed from June through September 2020. In October 2020, the number of SARS-CoV-2 infections rose again, marking the start of the second wave, which consisted of two parts: a first peak (October and November 2020), and a second peak (December 2020 and January 2021). The third wave started in February 2021, and progressed until June 2021 [2].

Nationwide infection control measures [3] were first introduced in March 2020, during the ‘intelligent’ lockdown of the Netherlands, which ended in June 2020. People were urged to stay home as much as possible, hand hygiene measures were encouraged, and the 1.5 m distance rule was introduced. Schools, restaurants, bars, theatres, ‘contact’ professions (e.g., hairdressers), and sport facilities were closed, and visits to nursing homes were not allowed. Availability of SARS-CoV-2 tests was limited to selected groups, such as health care workers. During the first part of the second wave, a ‘partial’ lockdown was introduced in October, allowing schools and sports facilities to remain open, but restricting group sizes outside and at home. Bars, restaurants, and entertainment facilities were closed. SARS-CoV-2-testing became widely available for the general public. During the second part of the second wave, The Netherlands went into a ‘strict’ lockdown in December 2020, which included the closure of schools and non-essential shops, and a curfew. Wearing non-medical facemasks was obligatory in indoor public spaces.

Although primary care services remained accessible throughout the first pandemic year, health care delivery and consultation behavior changed significantly for infectious as well as non-infectious illnesses [4,5,6,7]. After the first wave of the pandemic, we evaluated primary care presentation and antibiotic prescribing for common infection episodes in the Netherlands, and found a sharp decrease in respiratory and ear infections (after a sudden initial peak), with a concomitant decrease in antibiotic use [8]. The current analysis was initiated to evaluate how presentation and antibiotic use evolved after the first wave, having obtained the data from the entire first pandemic year. The intensity of infection control measures, the public’s compliance with them, and the social climate and awareness, have been changing constantly after the first wave. Therefore, the aim of our study is to determine how these successive phases in the full first pandemic year impacted on consulting and antibiotic prescribing for common infections in Dutch primary care, with a focus on the second and start of the third wave. Evaluating how consultation and antibiotic prescribing have been impacted by new contexts may provide clues on how to appropriate patients’ and health care providers’ behavior, with respect to health care use and antibiotic stewardship.

## 2. Results

### 2.1. Study Population

A total of 425,129 and 432,122 patients (49% male) were registered in the Julius General Practitioners’ Network in the year before and during the first COVID-19 pandemic year, respectively. The total numbers of consultations (face-to-face visit, via telephone, and e-mail) for common infections were 209,698 pre-pandemic, and 172,218 during the first pandemic year, which respectively related to 136,009 and 105,619 infectious disease episodes (sex and age distributions within the cohort and episodes can be found in Supplementary Tables S1–S3). The mean number of contacts per episode was 1.54 before and 1.63 during the pandemic.

### 2.2. Number of Infectious Disease Episodes for the Four Common Infection Types

The total number of infectious disease episodes was lower during the first pandemic year as compared to the pre-pandemic year (Table 1). The decline was largest for presentation of gastrointestinal infection episodes (RR, 0.58; CI, 0.56 to 0.60), and smallest for urinary tract infections (RR, 0.87; CI, 0.85 to 0.88).

**Figure 1:**
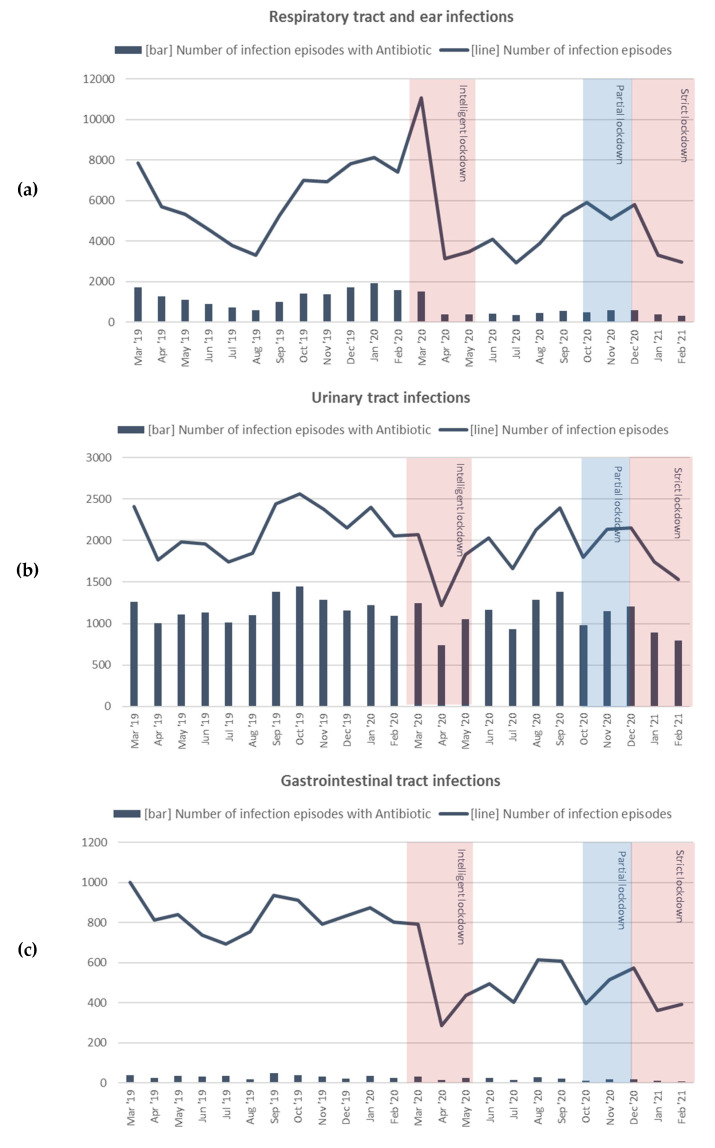

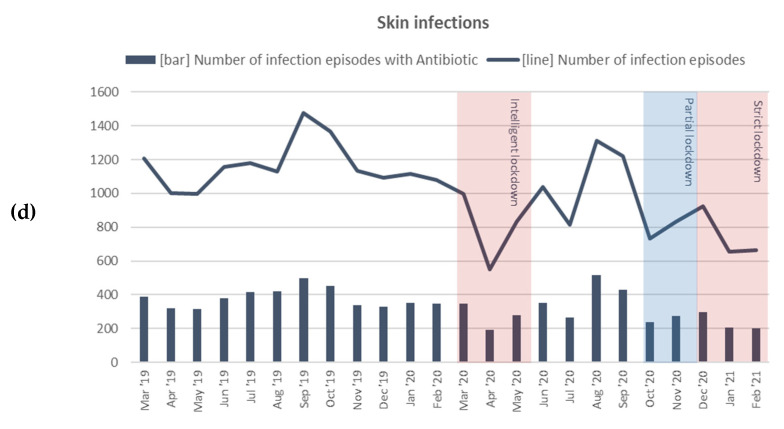
Number of episodes and episodes with antibiotic prescription for (**a**) respiratory tract/ear (**b**) urinary tract (**c**) gastrointestinal, and (**d**) skin infections over time per month, from March 2019 to February 2021.

Focusing on the number of episodes over time (Figure 1), a distinct peak in respiratory tract/ear infections was observed at the start of the COVID-19 pandemic. This peak was higher than the seasonal winter peak in the pre-pandemic year. After this initial peak, a sharp decline set in, resulting in fewer episodes at the end of the first wave, as compared to the same months in the previous year. In the summer period of 2020, the presentation of respiratory tract/ear episodes rose again, similar to the course in 2019. However, after October 2020, no usual winter peak developed. At the start of the ‘strict’ lockdown (second part of the second wave, December 2020 to January 2021), presentation decreased further. Contrary to the pattern seen during the first wave, this decline was not preceded by an initial increase (peak).

For urinary tract infections, an overall decline in the number of presented episodes was found, largely determined by the temporary decline during the first pandemic weeks. The first pandemic wave and ‘intelligent’ lockdown were associated with a decline in the numbers of gastrointestinal and skin infection episodes. Presentation did rise during the second wave, but remained below the levels in the pre-pandemic year. For skin infections, there was a peak during the period of low COVID-19 prevalence (June–September 2020), resembling the summer peak in the pre-pandemic year. During the second lockdown, the number of episodes for skin infections seemed to slightly decrease again.

### 2.3. Number of Infectious Disease Episodes with Antibiotics

Overall, episodes of common infection episodes treated with antibiotics declined during the first pandemic year (RR, 0.66; CI 0.65 to 0.67), mainly attributed to a decline in respiratory tract/ear infection episodes (RR, 0.41; CI, 0.40 to 0.42), as shown in Table 1.

The peak and subsequent fall in presentation of respiratory tract/ear infection episodes at the start of the pandemic coincided with a very sharp and persistent decrease in episodes treated with antibiotics, which lasted throughout the entire first pandemic year (Figure 1). The number of episodes treated with macrolides did not increase (Supplementary Figure S1).

The decline in presentation of urinary tract and skin infection episodes at the start of the pandemic also coincided with a temporal decline in the number of episodes with antibiotic prescription, which is similar to the pattern seen during the ‘strict’ lockdown period of the second wave.

### 2.4. Antibiotic Prescription Rate

**Figure 2:**
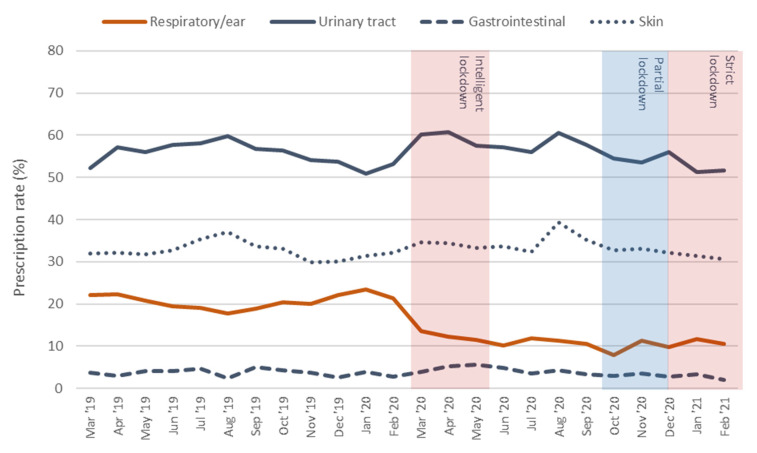
Course of the prescription rate (%) for four common infection types over time per month.

The proportion of episodes in which antibiotics were prescribed declined from 28% pre-pandemic to 24% in the first pandemic year, largely due to a 10% decline in the prescription rate for respiratory tract/ear infections (Table 2). The antibiotic prescription rates remained stable throughout all pandemic months for urinary tract, gastrointestinal, and skin infections (Figure 2). A marked decrease in prescription rate for respiratory tract/ear infections was apparent at the start of the pandemic, which continued to be lower during the successive waves.

### 2.5. Respiratory Tract/Ear Infection Episodes and Antibiotic Prescribing per Age Group

During the pandemic, fewer episodes for respiratory tract/ear infection were observed in all age categories, which was most pronounced in the young and eldest (Table 3; 0–12: RR, 0.50 (CI, 0.49 to 0.51); >65: RR, 0.72 (CI, 0.70 to 0.74)). The antibiotic prescription rate fell in all age categories, from 22% to 14% in the youngest group, and from 26% to 15% in the oldest group (Table 3). Supplementary Figure S2 shows the number of respiratory/ear infection episodes in total and with an antibiotic prescription over time per age group. In all patients ≥13 years of age, the pattern of episodes over time was similar to the pattern in Figure 1a. For young children, the natural pre-pandemic winter peak was in December, with higher presentation than observed during any of the pandemic waves. In contrast to the pattern in other age groups, no additional peak in presentation was seen during the initial weeks of the pandemic. No winter peak is observed during the course of the second wave of the pandemic.

### 2.6. Complications

Inappropriate withholding of antimicrobial treatment for bacterial infections may lead to an increase in complications. Registration of episodes indicative of complications from respiratory tract/ear and urinary tract infections remained similar or even decreased in the complete first pandemic year as compared to the previous year (Table 4; pyelonephritis: RR, 1.00 (CI, 0.88 to 1.14, *p* = 0.99); pneumonia: RR, 0.36 (CI, 0.34 to 0.38, *p* < 0.05); mastoiditis: RR, 0.56 (CI, 0.30 to 1.04, *p* = 0.07)). Temporal increases in complications were not detected during the pandemic waves (data not shown).

### 2.7. Remote Consultations

Part of the infectious disease episodes consisted of telephone consultations only, without face-to-face contact. The proportion of episodes with telephonic consultations only for the four common infection types increased during the pandemic (from 10.3% to 35.9%). The largest increase was seen for respiratory/ear infections (from 11.1% pre-pandemic to 43.7%), with peaks mid-first wave (April 2020, 50.6%) and during the first part of the second wave (October 2020, 50.2%, data not shown). The proportion of remote consultation for urinary tract episodes increased slightly during the pandemic (from 14.4% to 22.2%), and remained constant throughout the pandemic waves.

## 3. Discussion

### 3.1. Main Findings

Our observational cohort study reveals that after the first wave of the COVID-19 pandemic, GP consultation for common infection types remained lower during the full first pandemic year, in comparison with the pre-pandemic year. The decreased antibiotic prescribing for respiratory tract/ear infections which we had observed during the first wave continued into the second wave and the first part of the third wave, resulting in a year-lasting decrease in antibiotic prescribing. Prescription rates for other common infections remained stable.

When looking at the course of respiratory tract/ear infections after the first wave, the number of episodes for which GPs were consulted rose again over the summer, until the start of the second wave (October 2020). Possibly, this temporary rise was due to the absence of infection prevention measures. From October on, however, the usual winter peak did not develop. The ‘intelligent’ lockdown started, and presentation remained lower than in the previous winter. This could be attributed to a change in transmission of respiratory pathogens other than SARS-CoV-2 or changes in consultation behaviour; patients were urged to self-manage uncomplicated conditions, and remained afraid to contract COVID-19 in the doctor’s office. At the start of the ‘strict’ lockdown (December 2020 to January 2021), levels decreased even further, reaching a level similar to the first wave and lockdown.

Contrary to the pattern seen during the first wave, the decline during the second lockdown was not preceded by an initial increase (peak). During the first wave this peak might be explained by increased consultation behaviour, due to increased transmission in combination with COVID-19 concerns, fear, and ignorance at the start of the pandemic. The latter might have been less prominent during the second lockdown, when everyone was more familiar with the COVID-19 situation.

The presentation of gastrointestinal and skin infection episodes followed a similar pattern as the respiratory tract/ear infections. The ‘intelligent’ lockdown during the second wave was also associated with a decline in the numbers of gastrointestinal and skin infection episodes, possibly for the same reasons of transmission and behaviour changes. At no point during the first pandemic year did respiratory/ear, gastrointestinal, or skin infections recoil to pre-pandemic levels. On the contrary, presentation for urinary tract infections fluctuated during both the pandemic and the pre-pandemic year, and a sustained lower level was not observed as clearly.

When evaluating antibiotic prescribing during the first full pandemic year, we anticipated finding temporary increases for respiratory tract infections. Our findings show, however, that GPs did not prescribe more antibiotics at any time during periods of high COVID-19 prevalence. Furthermore, despite initial reports of a potential benefit of macrolide antibiotics for COVID-19 [9,10,11], macrolide prescribing did not increase. Moreover, the pandemic caused an overall significant decline in antibiotic prescribing for respiratory tract infections, more than any known intervention to promote antibiotic stewardship [12,13]. This lower level of prescribing could be a consequence of many years of antibiotic stewardship efforts undertaken in both primary and hospital care, with extensive education for both physicians and patients concerning rational use of antibiotics. The Netherlands ranks amongst the lowest when comparing antibiotic prescribing with other European countries [14]; our data reassure that antibiotic stewardship has not been neglected in the Netherlands because of the COVID-19 pandemic.

A potential downside of reduced antibiotic prescribing is that inappropriate withholding of antimicrobial treatment for bacterial infections may lead to an increase in complications. Fortunately, like in the first wave, we did not see an increase in complications during the rest of the first pandemic year. Furthermore, inappropriate withholding of antibiotics could lead to more hospitalisation and antimicrobial treatment in secondary care. When looking at antibiotic prescribing in hospitals (expressed in DDD/1000 inhabitant-days) the use of antibiotics routinely prescribed for respiratory tract infections (all types of penicillins and cephalosporins) decreased in 2020 as compared to 2019 and 2018 (unpublished data of the SWAB, Dutch Working Party on Antibiotic Policy).

### 3.2. Comparison with Other Literature

A prospective audit survey of consultation and management characteristics of patients with respiratory tract infections early in the first pandemic wave in 16 European countries showed that the proportion of patients prescribed antibiotics varied considerably between countries, and was generally lower during the pandemic as compared with the months before, except in Greece, Poland, and United Kingdom (UK) [15].

For the first pandemic wave, similar decreases in presentation and antibiotic prescribing for respiratory tract infections were found in a population-based cohort study in the UK, although in the UK, the initial increase in presentation was temporarily accompanied by an increase in antibiotic prescribing [16]. In Wales, the pandemic resulted in a significant reduction in dispenses of antibiotics commonly used for treatment of respiratory tract infection in the first wave, whereas dispensing of antibiotics primarily used for urinary tract and skin infections remained stable like in our study [17]. A study from northwest London included the second wave, and showed a sustained reduction in antibiotic prescribing after the first wave, similar to our findings [18].

In our study we did a sub-analysis looking at the prescription rate for respiratory tract and ear infection episodes in young children, and showed that the prescription rate remained lower during the full first pandemic year. A Dutch cohort study focusing specifically on childhood otitis media during the first pandemic year found that GP consultation declined, but antibiotic prescription rates remained similar [19]. The decrease found in our study may therefore be explained by a decrease in antibiotic prescribing for respiratory tract infections other than otitis media in this age group.

To our knowledge, no study so far has reported registrations of potential complications of reduced antibiotic prescribing in primary care.

### 3.3. Strengths and Limitations

Major strengths of our study are the large sample size using well-documented electronic routine care data, and the longitudinal nature of the study. This enabled us to compare the study population within the same practices during two full years.

Some limitations deserve further discussion. First, we were unable to capture data on out-of-hours or hospital care; results regarding complications should therefore be interpreted with caution. Second, the specific ICPC sub-code for confirmed COVID-19 was introduced in November 2020 (R83.3 SARS-CoV-2 (COVID-19)), and although the code was included in our data under the general code (R83 Other Respiratory Tract Infections), we were unable to specifically monitor the use of this code over the course of the full first pandemic year. Moreover, the vaccination status of patients was not taken into account; the vaccination campaign started in January 2021, and the general public was not vaccinated until after the current study period. It would be interesting to monitor consultation behaviour and antibiotic prescribing for respiratory tract infections in relation to vaccination status in future research. Lastly, conclusions of causality of the pandemic, presentation and antibiotic prescribing cannot be made using routine care data. The pandemic triggered changes in clinical guidelines, clinical practice, infection control, and individual and social behaviour. It is not possible to ascertain from this study which of these pandemic-induced changes were most influential.

### 3.4. Implications for Further Research

To ascertain lessons learned from the exceptional circumstances induced by the pandemic, various elements that may have influenced patient presentation and antibiotic prescribing warrant further investigation. First, the generic infection control measures, such as hand hygiene, may have limited the spread of communicable diseases [20], and thereby the need for consultation. It would be interesting to evaluate whether some of the generic infection control measures will have a sustained effect in post-pandemic years. Second, although primary care services remained accessible during the pandemic in the Netherlands, patients may have felt a higher threshold for visiting their GP, especially early in the pandemic, when patients were afraid to contract COVID-19 in the doctor’s office and the perceived burden on health care services was highest. Additionally, GPs and practice assistants may have felt more assertive in holding off consultations for uncomplicated conditions. This would explain the decrease in all types of infection episodes during the first wave, and also to a lesser extent later. This is unlikely to be the only explanation. If ‘consultation threshold’ would be the only factor involved, we would expect the antibiotic prescription rate to increase, due to a different case-mix in patients, with only more severely ill patients presenting to their GP. Such an increase in prescription rate was not observed for any of the infection types. Future research could aim to monitor how and why consultation behaviour and antibiotic prescribing develop in relation to SARS-CoV-2 occurrence, perhaps pinpointing more precisely, for example by using qualitative research methods, what is at the root of these changing patterns.

The decrease in antibiotic prescribing for respiratory tract infections may have been due to the widespread narrative of viral respiratory illness during the pandemic. GPs might have more confidently refrained from antibiotic therapy as patients were becoming more familiar with the viral origin of illness. The conversation in the GPs’ office about antibiotic therapy for respiratory disease might have been facilitated by the outbreak of a viral respiratory pathogen, resulting in more judicious antibiotic prescribing. The prescription rates for urinary tract, gastrointestinal, and skin infections remained stable over the course of the pandemic waves. These infections are mainly of bacterial origin, and thereby influenced by the pandemic and educational aspects related to self-limiting viral illness to a much lesser extent. It would be interesting to determine whether the viral narrative did indeed influence communication between GPs and patients and to learn whether and how this influenced prescribing decisions, by using qualitative research.

A final recommendation for future quantitative research would be to obtain more robust routine care data regarding complications and hospital referral during periods with reduced antibiotic prescribing.

## 4. Materials and Methods

### 4.1. Design and Study Population

This retrospective observational cohort study was conducted using pseudonymized routine health care data from the Julius General Practitioners’ Network (JGPN). The network covers 84 general practices providing care during office hours in Utrecht and the surrounding areas. Participating practices and their registered patients (approximately 370,000 individuals) are representative of the Dutch population as a whole [21].

All patients registered March 2019 through February 2020 (pre-COVID-19 pandemic) and/or March 2020 through February 2021 (during the COVID-19 pandemic first year) were included.

### 4.2. Data Extraction

General practitioners from the JGPN Network routinely register International Classification of Primary Care (ICPC) codes as part of electronic record keeping [21]. From the database, consultations for respiratory tract/ear, urinary tract, gastrointestinal, and skin infections were extracted using the ICPC codes listed in Appendix A (Table A1). The Dutch College of GPs advised GPs to use general ICPC codes for consultations about patients at risk for COVID-19, or for consultations about general COVID-19 concerns not related to an acute infection (A27 fear for other disease; A29 other symptoms/complaints), which were not included in the extraction.

ICPC codes from the same category, registered within 28 days after the initial consultation, were combined into infectious disease episodes. A new disease episode was constructed after 28 days without any infection related consultation in the same category.

The patient’s age, the episode start and stop dates, the number and type of consultations and antibiotic prescriptions were captured for each disease episode. The registration of the type of consultation may have been subject to change over the course of the study period, as long telephonic consultations may have been registered as face-to-face consultations more frequently during the pandemic compared to the year before.

Antibiotic prescriptions within two days before and after the start and stop dates of the episode were included, since individual consultations and prescriptions are not directly linked in the database.

### 4.3. Analyses

We calculated three main parameters: (1) the total number of GP-registered infectious disease episodes; (2) the absolute number of episodes treated with at least one antibiotic; and (3) the antibiotic prescription rate, i.e., the proportion of episodes treated with at least one antibiotic. Data were analysed for the four common infection types (respiratory tract/ear, urinary tract, gastrointestinal, skin) separately, compared between two time periods (pre-COVID-19-pandemic, March 2019 through February 2020, and during the first pandemic year, March 2020 through February 2021), as well as over time.

Differences in numbers of infectious disease episodes and differences in numbers of episodes treated with antibiotics between the two time periods were expressed as relative risks with accompanying 95% confidence intervals (CI). Statistical differences were determined using the chi-squared test. In calculating the relative risks, infection episodes were used as events and non-events were calculated by subtracting the number of episodes from the total number of registered patients. All statistical analyses were performed with SPSS (version 26.0, Chicago, IL, USA) and MedCalc for Windows, version 19.4 (MedCalc Software, Ostend, Belgium).

The course of the main parameters over time was determined per month for each infection type. Additionally, respiratory tract/ear infections outcomes were determined separately per age category (ages 0–12, 13–40, 41–65, >65). The numbers of respiratory tract/ear episodes with remote consultations only were analysed separately, as well as the specific ICPC codes for complications.

## 5. Conclusions

The COVID-19 pandemic led to a reduction in respiratory tract/ear infectious disease episodes for which a GP was consulted in the Netherlands. The start of the pandemic marked a sharp decline in antibiotic prescribing for respiratory tract/ear infections, resulting in a year-lasting decrease in antibiotic prescription rate (from 21% to 11%). Prescription rates for other common infections remained stable. The COVID-19 pandemic did not nullify antibiotic stewardship in the Netherlands, and may have opened the door to more judicious use of antibiotics for respiratory tract infections by shifting the narrative to the widespread viral origin of respiratory tract infection.

## Figures and Tables

**Table 1:** Numbers of disease episodes per infection type in total and with antibiotic prescription for respiratory/ear, urinary tract, gastrointestinal and skin infections, pre-pandemic and in the first pandemic year.

	Episodes			Episodes with Antibiotics		
	Pre- Pandemic	Pandemic		Pre- Pandemic	Pandemic	
	*n*	*n*	RR (CI)	*n*	*n*	RR (CI)
**Respiratory tract/ear**	73,089	56,875	0.77 (CI 0.76 to 0.77) *	15,335	6358	0.41 (CI 0.40 to 0.42) *
**Urinary tract**	25,703	22,690	0.87 (CI 0.85 to 0.88) *	14,216	12,815	0.89 (CI 0.87 to 0.91) *
**Gastrointestinal**	9990	5870	0.58 (CI 0.56 to 0.60) *	374	223	0.59 (CI 0.50 to 0.69) *
**Skin**	13,943	10,580	0.75 (CI 0.73 to 0.77) *	4558	3592	0.78 (CI 0.74 to 0.81) *
**Total**	122,725	96,015	0.77 (CI 0.76 to 0.78) *	34,483	22,988	0.66 (CI 0.65 to 0.67) *

Total numbers of patients registered: 425,129 pre-pandemic and 432,122 during the pandemic.* Values with a significance level *p* < 0.05.

**Table 2:** Prescription rates for common infections pre-pandemic and during the complete first pandemic year.

	Pre-Pandemic	Pandemic	Difference (CI)
	%	%	
**Respiratory tract/ear**	21%	11%	−10% (CI −10.4 to −9.6%) *
**Urinary tract**	55%	56%	+1% (CI 0.1% to 1.9%) *
**Gastrointestinal**	4%	4%	+0% (CI −0.6% to 0.6%)
**Skin**	33%	34%	+1% (CI −0.2% to 2.2%)
**Total**	28%	24%	−4% (CI −4.4 to −3.6%) *

* Values with a significance level *p* < 0.05.

**Table 3:** Relative risks of respiratory/ear infection episodes (total and with antibiotic prescription) and prescription rates pre-pandemic and during the first pandemic year, per age category.

Age Group	Episodes	Episodes with Antibiotic	Prescription Rate	
			Pre-Pandemic	Pandemic
	RR (CI)	RR (CI)	%	%
**0–12**	0.50 (CI 0.49 to 0.51)	0.33 (CI 0.31 to 0.35)	22%	14%
**13–40**	0.87 (CI 0.85 to 0.88)	0.42 (CI 0.40 to 0.44)	18%	9%
**41–65**	0.96 (CI 0.95 to 0.98)	0.49 (CI 0.46 to 0.52)	20%	10%
**>65**	0.72 (CI 0.70 to 0.74)	0.42 (CI 0.39 to 0.44)	26%	15%
**Total**	0.77 (CI 0.76 to 0.77)	0.41 (CI 0.40 to 0.42)	21%	11%

Relative risks and differences in pre-pandemic and pandemic prescription rates are all significant (*p* < 0.05). Total numbers of patients registered (pre-pandemic and during the pandemic, respectively), 0–12: 67,263 and 67,159; 13–40: 177,829 and 181,579; 41–65: 126,919 and 128,638; >65: 53,114 and 54,740.

**Table 4:** Numbers of registered diagnoses indicating complications pre-pandemic and during the first pandemic year.

	Pre-Pandemic	Pandemic	
	*n*	*n*	RR (CI)
**Pyelonephritis**	460	467	1.00 (CI 0.88 to 1.14)
**Pneumonia**	3934	1423	0.36 (CI 0.34 to 0.38) *
**Mastoiditis**	28	16	0.56 (CI 0.30 to 1.04)

* Values with a significance level *p* < 0.05.

## Data Availability

Data are not publicly available due to ethical and legal restrictions.

## Supplementary Materials

The following are available online at www.mdpi.com/article/10.3390/antibiotics10121521/s1. Table S1: Age and sex distribution Julius General Practitioners’ Network cohort pre-pandemic and in the first pandemic year. Table S2: Age distribution for respiratory/ear, urinary tract, gastrointestinal, and skin infection episodes, pre-pandemic and in the first pandemic year. Table S3: Sex distribution for respiratory/ear, urinary tract, gastrointestinal, and skin infection episodes, pre-pandemic and in the first pandemic year. Figure S1: Macrolide prescription for respiratory infections over time. Figure S2: Number of respiratory and ear infection episodes and episodes with antibiotic prescription over time per age category.

## Appendix A

**Table A1:** ICPC codes used to group consultations into episodes (respiratory tract/ear, urinary tract, gastrointestinal and skin infection episodes).

ICPC Chapter	
**H—Ear** **R—Respiratory tract**	H71 acute otitis media H99 ear/mastoid disease H01 earache H04 ear discharge H70 otitis externa H72 serous otitis media H74 chronic otitis media/other otitis H74.01 chronic otitis media H74.02 mastoiditis R21 throat symptoms R22 tonsil symptoms R72 strep throat R74 acute upper RTI R76 tonsillitis/tonsillar abscess R76.01 tonsillitis R76.02 tonsillar abscess R77 laryngitisR80 influenza R83 other RTI (including COVID-19) R05 cough R09 sinus symptoms R75 sinusitis R78 bronchitis R81 pneumonia R82 pleurisy R71 whooping cough R70 tuberculosis
**U—Urinary tract**	U01 dysuria U02 urgency U71 cystitis U70 pyelonephritis Y73 prostatitis
**D—Gastrointestinal**	D11 diarrhoea D70 gastrointestinal infection D73 gastroenteritis D22 parasites D92 diverticular disease
**S—Skin**	S09 infected finger/toe S10 boil/carbuncle S11 skin infection post-traumatic S76 other skin infection S84 impetigo
