# Supplementary material for: Common Infections and Antibiotic Prescribing during the First Year of the COVID-19 Pandemic: A Primary Care-Based Observational Cohort Study
Source: Antibiotics (Basel). 2021 Dec 13;10(12):1521. doi: 10.3390/antibiotics10121521 (PMC8698485; doi:10.3390/antibiotics10121521)
Supplement: Supplementary file 1 [file antibiotics-10-01521-s001.zip › antibiotics-1466448-supplementary.pdf]

Supplementary Materials

Common Infections and Antibiotic Prescribing during the First Year of the COVID-19 Pandemic: A Primary Care-Based Observational Cohort Study

Josi A. Boeijen *, Alike W. van der Velden, Saskia Hullegie, Tamara N. Platteel, Dorien L. M. Zwart, Roger A. M. J. Damoiseaux, Roderick P. Venekamp and Alma C. van de Pol

Julius Center for Health Sciences and Primary Care, University Medical Center Utrecht, Utrecht University, Heidelberglaan 100, 3584 CX Utrecht, The Netherlands; a.w.vandervelden@umcutrecht.nl (A.W.v.d.V.); s.hullegie@umcutrecht.nl (S.H.); T.N.Platteel-3@umcutrecht.nl (T.N.P.); D.Zwart@umcutrecht.nl (D.L.M.Z.); R.A.M.J.Damoiseaux@umcutrecht.nl (R.A.M.J.D.); R.P.Venekamp@umcutrecht.nl (R.P.V.); A.C.vandePol-11@umcutrecht.nl (A.C.v.d.P.)

* Correspondence: j.a.boeijen@umcutrecht.nl

Table S1. Age and sex distribution Julius General Practitioners' Network cohort pre-pandemic and in the first pandemic year.

Age Groups		Male *n* = (%)	Female *n* = (%)	Other *n* = (%)	Total *n* = (%)
0–12	Pre-pandemic	34,324 (51.0)	32,938 (49.0)	1 (0.0)	67,263 (100)
	Pandemic	34,288 (51.1)	32,870 (48.9)	1 (0.0)	67,159 (100)
13–40	Pre-pandemic	84,519 (47.5)	93,307 (52.5)	3 (0.0)	177,829 (100)
	Pandemic	86,412 (47.6)	95,164 (52.4)	3 (0.0)	181,579 (100)
41–65	Pre-pandemic	63,782 (50.3)	63,137 (49.7)	0 (0.0)	126,919 (100)
	Pandemic	64,598 (50.2)	64,040 (49.7)	0 (0.0)	128,638 (100)
>65	Pre-pandemic	23,895 (45.0)	29,218 (55.0)	1 (0.0)	53,114 (100)
	Pandemic	24,737 (45.2)	30,002 (54.8)	1 (0.0)	54,740 (100)
Unknown Age	Pre-pandemic	4 (100)	0 (0.0)	0 (0.0)	4 (100)
	Pandemic	6 (100)	0 (0.0)	0 (0.0)	6 (100)
Total	Pre-pandemic	206,524 (48.6)	218,600 (51.4)	5 (0.0)	425,129 (100)
	Pandemic	210,041 (48.6)	222,076 (51.4)	5 (0.0)	432,122 (100)

Table S2. Age distribution for respiratory/ear, urinary tract, gastrointestinal and skin infection episodes, pre-pandemic and in the first pandemic year.

		Median Age (IQR)	Age Groups 0–12 *n* = (%)	13–40 *n* = (%)	41–65 *n* = (%)	>65 *n* = (%)	Total *n* = (%)
**Respiratory tract/ear**	**Pre-pandemic**	34 (8-56)	20,588 (28.2)	22,209 (30.4)	18,714 (25.6)	11,578 (15.8)	73,089 (100)
	**Pandemic**	39 (21-57)	10,265 (18.0)	19,695 (34.6)	18,292 (32.2)	8623 (15.2)	56,875 (100)
**Urinary tract**	**Pre-pandmic**	50 (29-70)	1608 (6.3)	8837 (34.4)	7252 (28.2)	8006 (31.1)	25,703 (100)
	**Pandemic**	51 (30-72)	1430 (6.3)	7559 (33.3)	6286 (27.7)	7415 (32.7)	22,690 (100)
**Gastrointestinal**	**Pre-pandmic**	31 (8-60)	2910 (29.1)	3069 (30.7)	2018 (20.2)	1993 (19.9)	9990 (100)
	**Pandemic**	39 (16-66)	1380 (23.5)	1654 (28.2)	1366 (23.3)	1470 (25,0)	5870 (100)
**Skin**	**Pre-pandmic**	32 (11-55)	3678 (26.4)	4694 (33.7)	3256 (23.4)	2315 (16.6)	13,943 (100)
	**Pandemic**	35 (16-59)	2381 (22.5)	3523 (33.3)	2628 (24.8)	2048 (19.4)	10,580 (100)
**Total**	**Pre-pandmic**	36 (15-60)	28,784 (23.5)	38,809 (31.6)	31,240 (25.5)	23,892 (19.5)	122,725 (100)
	**Pandemic**	41 (23-61)	15,456 (16.1)	32,431 (33.8)	28,572 (29.8)	19,556 (20.4)	96,015 (100)

Abbreviations: IQR = interquartile range.

Table S3. Sex distribution for respiratory/ear, urinary tract, gastrointestinal and skin infection episodes, pre-pandemic and in the first pandemic year.

		**Male n = (%)**	**Female n = (%)**	**Other n = (%)**	**Total n = (%)**
**Respiratory tract/ear**	**Pre-pandmic**	30,924 (42.3)	42,165 (57.7)	0 (0.0)	73,089 (100)
	**Pandemic**	24,427 (42.9)	32,447 (57.0)	1 (0.0)	56,875 (100)
**Urinary tract**	**Pre-pandmic**	5178 (20.1)	20,525 (79.9)	0 (0.0)	25,703 (100)
	**Pandemic**	4537 (20.0)	18,153 (80.0)	0 (0.0)	22,690 (100)
**Gastrointestinal**	**Pre-pandmic**	4187 (41.9)	5803 (58.1)	0 (0.0)	9990 (100)
	**Pandemic**	2414 (41.1)	3456 (58.9)	0 (0.0)	5870 (100)
**Skin**	**Pre-pandmic**	6777 (48.6)	7165 (51.4)	1 (0.0)	13,943 (100)
	**Pandemic**	5062 (47.8)	5518 (52.2)	0 (0.0)	10,580 (100)
**Total**	**Pre-pandmic**	47,066 (38.4)	75,658 (61.6)	1 (0.0)	122,725 (100)
	**Pandemic**	36,440 (38.0)	59,574 (62.0)	1 (0.0)	96,015 (100)

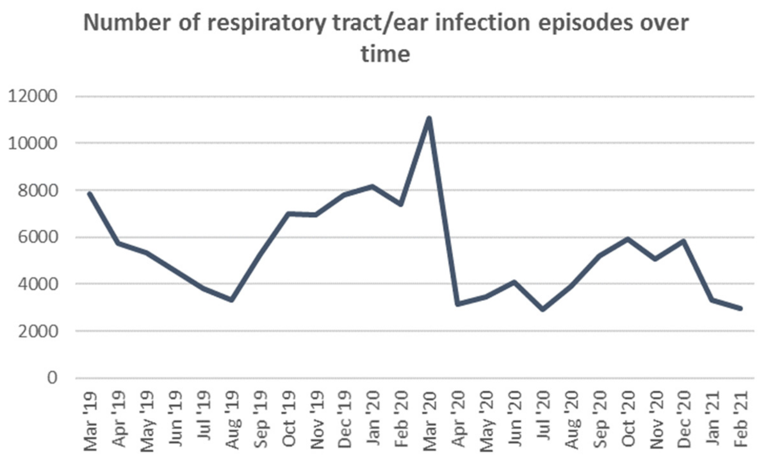

(**a**)

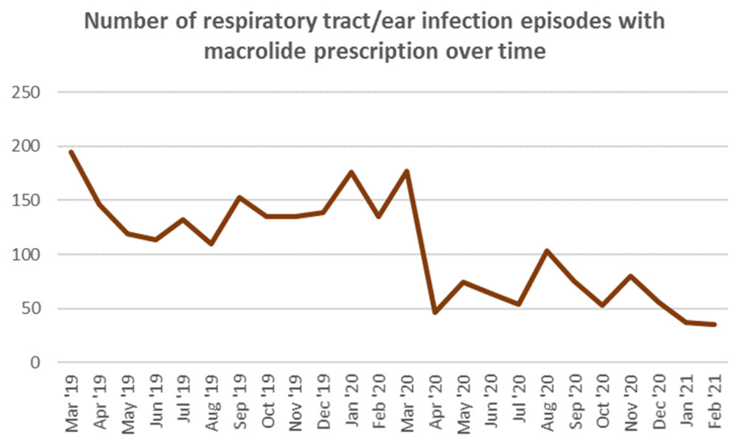

(**b**)

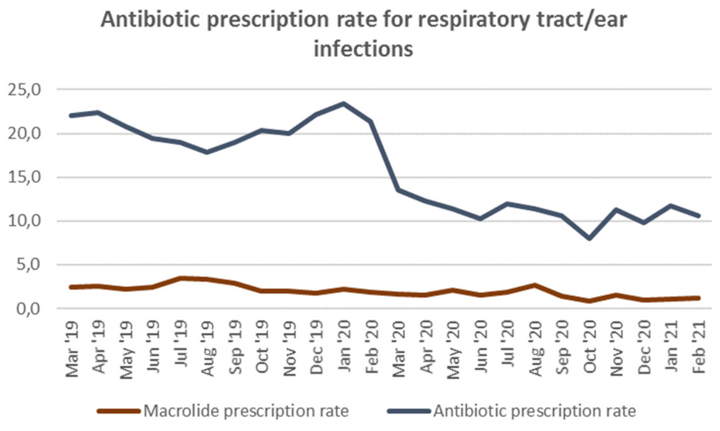

(**c**)

Figure S1. Macrolide prescription for respiratory infections over time: (**a**) number of respiratory tract/ear infection episodes over time; (**b**) number of respiratory tract/ear infection episodes with macrolide prescription over time; (**c**) antibiotic prescription rate for respiratory tract/ear infections.

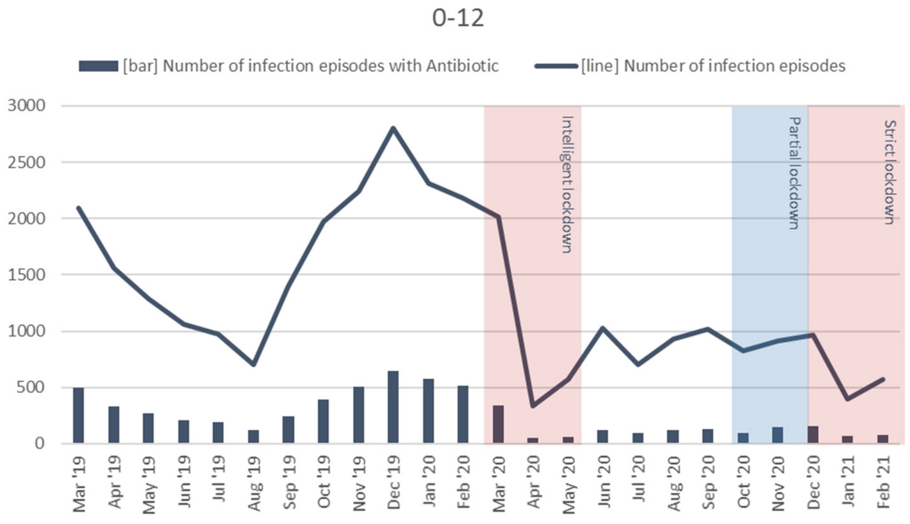
0-12
[bar] Number of infection episodes with Antibiotic
[line] Number of infection episodes
3000
2500
2000
1500
1000
500
0
Intelligent lockdown
Partial lockdown
Strict lockdown
Mar '19
Apr '19
May '19
Jun '19
Jul '19
Aug '19
Sep '19
Oct '19
Nov '19
Dec '19
Jan '20
Feb '20
Mar '20
Apr '20
May '20
Jun '20
Jul '20
Aug '20
Sep '20
Oct '20
Nov '20
Dec '20
Jan '21
Feb '21

(**a**)

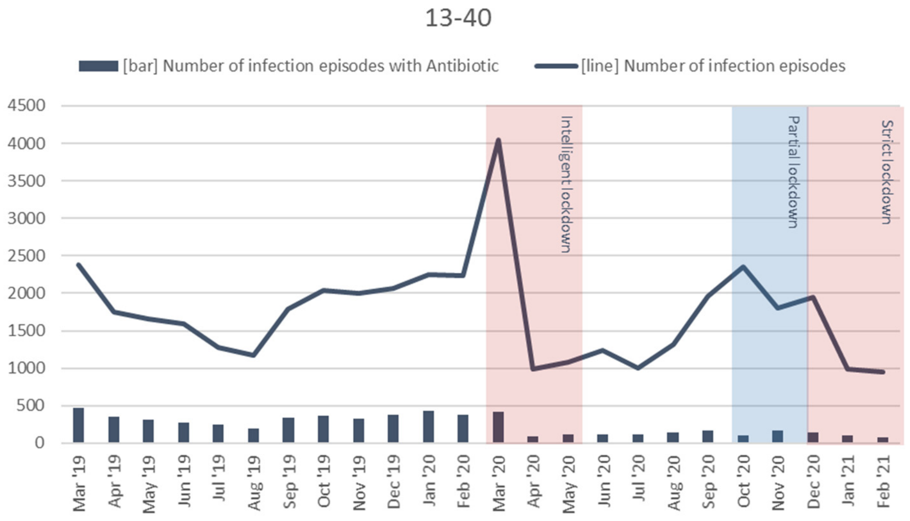
13-40
[bar] Number of infection episodes with Antibiotic
[line] Number of infection episodes
4500
4000
3500
3000
2500
2000
1500
1000
500
0
Intelligent lockdown
Partial lockdown
Strict lockdown
Mar '19
Apr '19
May '19
Jun '19
Jul '19
Aug '19
Sep '19
Oct '19
Nov '19
Dec '19
Jan '20
Feb '20
Mar '20
Apr '20
May '20
Jun '20
Jul '20
Aug '20
Sep '20
Oct '20
Nov '20
Dec '20
Jan '21
Feb '21

(**b**)

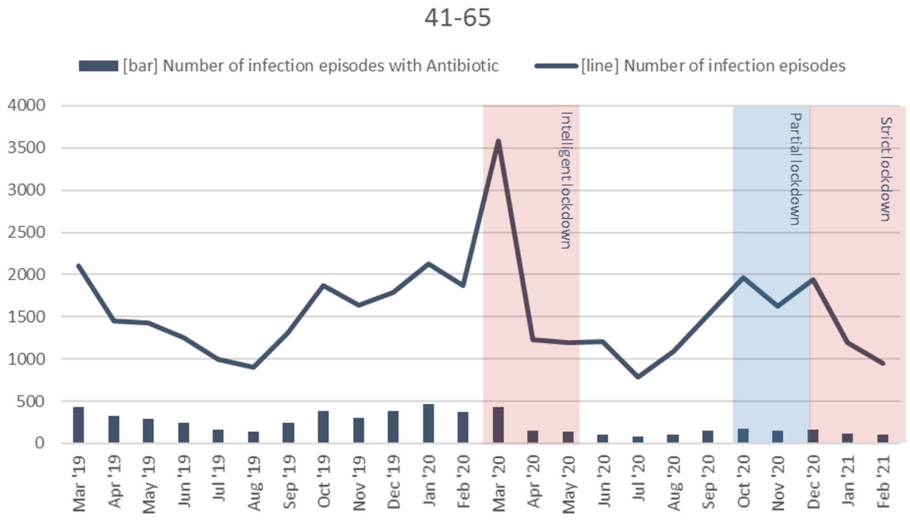
41-65
[bar] Number of infection episodes with Antibiotic
[line] Number of infection episodes
4000
3500
3000
2500
2000
1500
1000
500
0
Intelligent lockdown
Partial lockdown
Strict lockdown
Mar '19
Apr '19
May '19
Jun '19
Jul '19
Aug '19
Sep '19
Oct '19
Nov '19
Dec '19
Jan '20
Feb '20
Mar '20
Apr '20
May '20
Jun '20
Jul '20
Aug '20
Sep '20
Oct '20
Nov '20
Dec '20
Jan '21
Feb '21

(**c**)

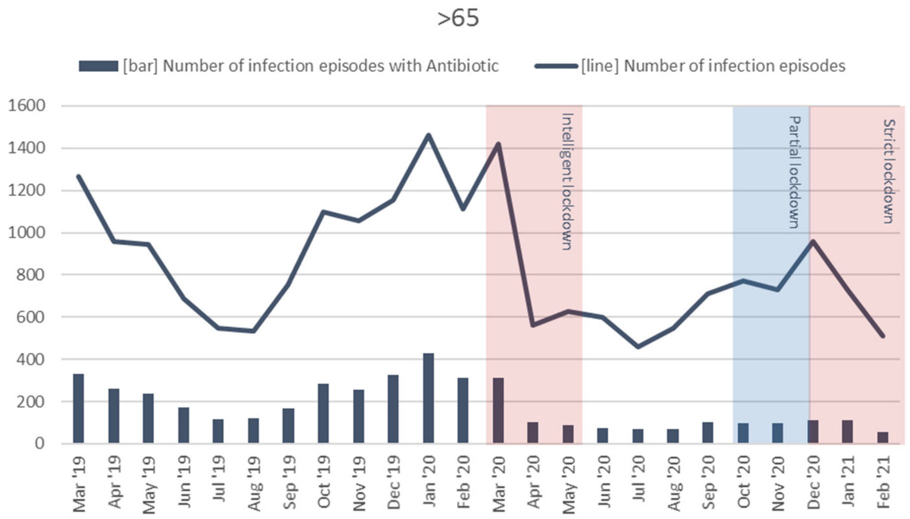

(**d**)

Figure S2. Number of respiratory and ear infection episodes and episodes with antibiotic prescription over time per age category: (**a**) 0–12, (**b**) 13–40, (**c**) 41–65, and (**d**) >65.
